# Long‐Term Risk of Clinically Significant Prostate Cancer in Biopsy‐Negative Patients With Baseline Biparametric Prostate MRI

**DOI:** 10.1002/jmri.29668

**Published:** 2024-11-27

**Authors:** Laura Parhiala, Juha Knaapila, Ivan Jambor, Janne Verho, Kari Syvänen, Hannu Aronen, Peter Boström, Otto Ettala

**Affiliations:** ^1^ Department of Urology University of Turku Turku Finland; ^2^ Department of Urology Turku University Hospital Turku Finland; ^3^ Department of Urology Tampere University Hospital Tampere Finland; ^4^ Department of Urology Kuopio University Hospital Kuopio Finland; ^5^ Department of Diagnostic Radiology University of Turku Turku Finland; ^6^ Medical Imaging Centre of Southwest Finland Turku University Hospital Turku Finland; ^7^ Enterprise Service Group—Radiology Mass General Brigham Boston Massachusetts USA

## Abstract

**Background:**

The long‐term prevalence of clinically significant prostate cancer (csPCa) in patients with initial negative prostate biopsy is unknown.

**Purpose:**

To investigate the rate of csPCa of men with initial negative biopsy.

**Study Type:**

Retrospective analysis of prospectively collected data.

**Population:**

A total of 197 men (mean age 63 years [SD ±6.98, range 29–79]) without csPCa on initial biopsy and available baseline biparametric prostate MRI (bpMRI).

**Field Strength/Sequence:**

3.0 T, turbo spin‐echo T2‐weighted (axial and sagittal) and three sets of diffusion‐weighted imaging using single‐shot spin‐echo planar imaging (5 b‐values 0–500 seconds/mm^2^; 2 b‐values 0 and 1500 seconds/mm^2^, and 2 b‐values 0 and 2000 seconds/mm^2^).

**Assessment:**

BpMRI was read using Prostate Imaging Reporting Data System (PI‐RADS) v2.1. Systematic or targeted biopsy results served as reference standard.

**Statistical Tests:**

Continuous variables were compared using Kruskal–Wallis rank sum test. Categorical variables were compared using either Fisher's exact test or Pearson's chi‐square test. Uni‐ and multivariate regression odds ratios (95% confidence interval) were used to study factors affecting csPCa being diagnosed during follow‐up. Time to diagnosis of csPCa is calculated using the Kaplan–Meier method.

**Results:**

Of 197 men, 74 (38%), 57 (29%), and 66 (34%) presented with PI‐RADS 1–2, 3, and 4–5 findings in the baseline bpMRI. During the median follow‐up of 52 months, 8.1%, 5.3%, and 18.2% of these men were diagnosed with csPCa, respectively. Baseline PI‐RADS finding was the only factor that associated with csPCa found during the follow‐up.

**Data Conclusion:**

Baseline bpMRI with PI‐RADS scores 1–3 and initial biopsies negative of csPCa had low rate of csPCa during follow‐up, which supports more conservative follow‐up for them but further research with longer follow‐up is warranted.

**Level of Evidence:**

3

**Technical Efficacy:**

Stage 2

European Association of Urology (EAU) guidelines strongly recommend to perform a prebiopsy multiparametric MRI (mpMRI) for men with a clinical suspicion of prostate cancer (PCa).^1^ This approach was shown to reduce the number of unnecessary biopsies (meaning negative prostate biopsies), decrease the detection of clinically nonsignificant PCa, and improve the detection of clinically significant PCa (csPCa). The use of intravenous contrast media and performing dynamic contrast‐enhanced MRI, as a part of mpMRI, increases the acquisition time and costs.^2^ In contrast to a standard mpMRI, biparametric MRI (bpMRI) is performed without contrast media, and therefore it is less time‐consuming and thus more cost‐effective, while it also has a comparable diagnostic performance compared to mpMRI.^3^, ^4^, ^5^, ^6^, ^7^, ^8^

In multiple studies, the rate of the International Society of Urological Pathology Gleason Grade Group (GGG) ≥2 PCa in systematic biopsies was shown to be very low in men with prebiopsy mpMRI or bpMRI findings of Prostate Imaging Reporting Data System (PI‐RADS) score of 1–2.^1^, ^2^, ^3^, ^4^, ^5^, ^6^ In contrast, in these studies, targeted biopsies of PI‐RADS 4–5 findings yielded mostly GGG ≥2 PCa.^1^, ^2^, ^3^, ^4^, ^5^, ^6^ The cases with a PI‐RADS score of 3 remain a diagnostic challenge. These MRI findings are traditionally seen as equivocal and the rate of GGG ≥2 varies widely between studies.^9^ In a meta‐analysis by Maggi et al which included 25 studies, the rate of GGG ≥2 in PI‐RADS 3 cases was 18.5% ranging from 3.4% to 47%.^10^ Therefore, EAU guidelines recommend biopsy in most PI‐RADS 3 findings despite the disadvantages of overdiagnosis.^1^

BpMRI as a triage test was shown to have the potential for ruling out csPCa, but there is relatively little evidence whether bpMRI PI‐RADS 1–2 or even PI‐RADS 1–3 findings predict a csPCa free status also in the long term.^5^, ^6^, ^11^, ^12^ Recent follow‐up studies have shown that the risk of being diagnosed with csPCa (GGG ≥ 2) after PI‐RADS 1–2 in baseline mpMRI within the next few years is under 6%.^13^, ^14^, ^15^, ^16^ Similar results were shown for men with PI‐RADS 3 findings, specifically the risk of being diagnosed with GGG ≥2 PCa during follow‐up range from 4% to 6.4% in men with a baseline mpMRI PI‐RADS score of 3.^15^, ^16^, ^17^, ^18^ However, these studies which reported the rate of incident of PCa in men with baseline PI‐RADS 3 findings were conducted using mpMRI, and therefore, it would be very interesting to find out if similar results can be obtained also with bpMRI. This is highly relevant since some of the PI‐RADS 3 lesions are upgraded to PI‐RADS 4 lesions due to contrast enhancement observed in sequences which are only performed in mpMRI and require an intravenous contrast media injection. Whether this influences the rate of incident of csPCa during long‐term follow‐up is not known.

The aim of the study was to investigate the rate of incident of GGG ≥2 PCa during long‐term follow‐up among men with PI‐RADS 3, PI‐RADS 1–2, and PI‐RADS 4–5 findings in baseline bpMRI and baseline biopsies negative of GGG ≥2 PCa.

## Materials and Methods

### Cohort

This retrospective study cohort consisted of men enrolled in three prospective and registered, single‐center IMproved PROstate cancer Diagnosis (IMPROD) bpMRI trials (IMPROD NCT01864135, IMPROD 2.0 NCT02844829, and PROMANEG NCT02388126) and one multicenter IMPROD bpMRI trial (Multi‐IMPROD NCT02241122), conducted between March 2013 and May 2017. Inclusion in all the trials were a suspicion of PCa based on an increased prostate‐specific antigen (PSA) (2.5–20 ng/mL) and/or an abnormal finding in a digital rectal examination. In the current study, exclusion criteria were 1) follow‐up at other centers and not available follow‐up data and 2) presence of csPCa (GGG 2–5) in the initial biopsies.

### Baseline MRI and Biopsy Protocols

The prebiopsy prostate bpMRI was performed with a 3.0 T scanner (3 T Verio, Siemens Healthineers, Erlangen, Germany) using surface coils. MRI protocol followed the IMPROD bpMRI protocol,^3^, ^4^, ^5^, ^19^, ^20^ with an adaptable version available at https://mrc.utu.fi/protocols/prostate. The protocol consists of turbo spin‐echo T2‐weighted imaging (axial and sagittal) and three separate diffusion‐weighted imaging acquisitions: 1) repetition time/echo time (TR/TE) 5543/80 msec, b‐values 0, 100, 200, 350, and 500 seconds/mm^2^, acquisition voxel size 2.0 × 2.0 × 3.0 mm^3^, no slice gaps (20); 2) TR/TE 5000/87 msec b‐values 0, 1500 seconds/mm^2^, acquisition voxel size 2.0 × 2.0 × 5.0 mm^3^, no slice gaps; and 3) TR/TE 5000/87 msec, b values 0, 2000 seconds/mm^2^, acquisition voxel size 2.0 × 2.0 × 5.0 mm^3^, no slice gaps. The overall scan time, including shimming and calibration, was 14–17 minutes. All imaging datasets were reported using IMPROD Likert bpMRI and PI‐RADS scoring system^3^ by a local radiologist (JV) with 1‐year degree of experience, and confirmed centrally by one designated central reader (IJ), 4 years of experience in prostate MRI at the beginning of the first trial in 2013, to guarantee reporting integrity before each biopsy procedure. Following completion of the trials, all the MRI studies were re‐reported using PI‐RADS version 2.1. The follow‐up MRIs were performed using the same bpMRI protocol and the same MRI scanner. In the current analysis a PI‐RADS version 2.1 scoring system was used since only very minor differences were present between prospectively assigned IMPROD bpMRI Likert scores and retrospectively assigned PI‐RADS version 2.1.^19^, ^21^

Depending on the prostate volume, 10‐ or 12‐core systematic biopsies were taken. If lesions with PI‐RADS 3–5 were present, two targeted biopsies per lesion were taken. In the IMPROD trial, only the dominant lesion was targeted (162/430, 38%). All baseline and follow‐up biopsies were taken via transrectal approach using cognitive MRI‐transrectal ultrasound targeting. The baseline biopsies were taken by a few specific urologists involved in the study. The follow‐up biopsies were taken by any urologists of the study center and based on clinical practice.

### Follow‐Up of the Study Population

Follow‐up of the study population with benign or GGG 1 PCa in initial biopsies was based on routine clinical practice: if presenting with a rising PSA or any other condition or symptoms suggesting csPCa, patients were referred back to the study center. Subsequent biopsies were taken as per normal clinical practice including a combination of systematic and targeted biopsies or systematic or targeted alone. There was no specific predesigned follow‐up plan for the study population.

### Data Collection

All the data were gathered retrospectively from prospective trials. Pseudonymized data was collected, managed, and stored in a REDCap electronic data capture tool.^22^, ^23^

### Statistical Analysis

Continuous variables are presented as medians (interquartile range [IQR]) and were compared using Kruskal–Wallis rank sum test. Categorical variables are presented as *N* (%) and were compared using either Fisher's exact test or Pearson's chi‐square test. Multivariate logistic regression analysis with odds ratios (95% CI) was used to investigate factors associated with the diagnosis of csPCa during follow‐up. Time to diagnosis of csPCa was analyzed using the Kaplan–Meier method.

### Study Hypothesis and Primary Outcome

The hypothesis of this study was that the rate of csPCa detected during the follow‐up would be significantly different depending on the PI‐RADS v2.1 initial score. The hypothesis was that with bpMRI PI‐RADS scores 1–2 and 3 in the baseline biopsies, the risk of being diagnosed with GGG ≥2 PCa during follow‐up time would be very low.

The primary outcome of this study is the incidence of csPCa (GGG ≥ 2) during the follow‐up in men with PI‐RADS 1–2, 3, and 4–5 findings in baseline bpMRI and without csPCa in initial biopsy.

## Results

**FIGURE 1:**
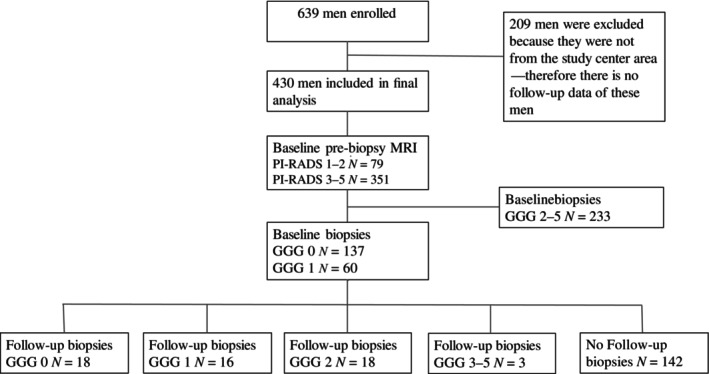
Flow chart of the study population.

From a total of 639 enrolled men, 209 (33%) were excluded due to clinical follow‐up outside of the study center referral area, and 233 (54%) were excluded due to csPCa (Fig. 1). A total of 430 men (mean age 65 ± SD 6.83 years) were included into the study, and 197 men (mean age 63 ± SD 6.98 years) had benign or GGG 1 PCa in initial biopsies. The study population in the IMPROD, multi‐IMPROD, and IMPROD 2.0 trials consisted of mostly biopsy‐naïve men; previous negative biopsies were taken from 11% (18/162), 0% (0/129), and 2% (1/55), respectively. In the PROMANEG trial, 94% (79/84) of men had previous negative biopsies and a prevailing clinical suspicion of csPCa. The baseline characteristics of the study cohort are summarized in Table 1.

**TABLE 1:** Characteristics of the Study Population

Variables	Baseline Benign or GGG 1 (*N* = 197)
Age (years)	63 (29–79)
PSA (ng/mL)	7 (5.6–9.5)
Free PSA/PSA ratio (%)	13 (6.6–18)
Prostate volume (mL)	42 (30–58)
PSA density (ng/mL^2^)	0.15 (0.10–0.23)
5‐Alpha reductase therapy (yes)	34 (17.3)
Previous procedures	
None	144 (73.0)
Prostate biopsies	49 (25)
Prostate surgery	4 (2)
Baseline bpMRI PI‐RADS score	
PI‐RADS 1–2	74 (37.6)
PI‐RADS 3	57 (28.9)
PI‐RADS 4–5	66 (33.5)

Data are mean ± standard deviation (range), median (interquartile range), or number (percentage).

GGG = Gleason Grade Group; bpMRI = biparametric MRI; PSA = prostate‐specific antigen; PI‐RADS = Prostate Imaging Reporting Data System v2.1.

Median follow‐up interval was 52 (IQR: 21–75) months in 197 patients with benign or GGG 1 PCa in the baseline biopsies. The more intensive follow‐ups using MRI and biopsy was performed in patients with high PI‐RADS scores on bpMRI: 44% (29/66) in patients with PI‐RADS 4–5 and 32% (18/57) in with PI‐RADS 3. Among patients with PI‐RADS 1–2, only 11% (8/74) underwent MRI and prostate biopsy were taken only from 20% (15/74) during the follow‐up (Table 2). In patients with the same PI‐RADS score, the follow‐up of men with GGG 1 cancer in baseline biopsies was more intensive on all measures compared to those with GGG 0 (Table S1 in the Supplemental Material); follow‐up time was longer; follow‐up PSAs were taken from every man at least once and more often during the follow‐up time and both follow‐up MRIs and biopsies were taken more frequently.

**TABLE 2:** Characteristics of Follow‐Up Procedures in Patients With No Cancer or GGG 1 Cancer at Baseline Biopsies

Baseline MRI	PI‐RADS 1–2 (*N* = 74)	PI‐RADS 3 (*N* = 57)	PI‐RADS 4–5 (*N* = 66)	*P*
Median follow‐up period (months)	40 (18–69)	45 (23–74)	66 (28–80)	0.040
Patients with follow‐up PSA^a^	64 (87.0)	55 (97.0)	63 (96.0)	0.062
Median number of follow‐up PSA	3 (1–8)	6 (3–11)	9 (4–14)	<0.001
Patients with follow‐up MRI	15 (20.0)	18 (32.0)	29 (44.0)	0.011
Patients with follow‐up biopsy	8 (11.0)	18 (32.0)	29 (44.0)	<0.001

Data are median (interquartile range) or number (percentage).

PSA = prostate specific antigen; GGG = Gleason Grade Group; PI‐RADS = Prostate Imaging Reporting Data System.

^a^ Follow‐up PSA was obtained in all patients at once, but the data were not available in 15 patients.

During follow‐up, csPCa (GGG ≥ 2) was diagnosed in 8.1% (6/74) of patients with PI‐RADS 1–2 lesions and 5.3% (3/57) with PI‐RADS 3 lesions (Table 3). In patients with PI‐RADS 4–5 lesions, 18% (12/66) were diagnosed with csPCa, despite initial benign or GGG 1 PCa biopsy results. One patient in each PI‐RADS category was diagnosed with GGG ≥3 PCa.

**TABLE 3:** Outcomes of Follow‐Up MRI and Follow‐Up Biopsies in Patients With No Cancer or GGG 1 Cancer at Baseline Biopsies

Outcome	Baseline PI‐RADS 1–2 (*N* = 74)	Baseline PI‐RADS 3 (*N* = 57)	Baseline PI‐RADS 4–5 (*N* = 66)
Follow‐up MRI			
PI‐RADS 1–2	6 (8.1)	8 (14.0)	3 (4.5)
PI‐RADS 3	6 (8.1)	7 (12.3)	7 (10.6)
PI‐RADS 4–5	3 (4.1)	3 (5.3)	19 (28.8)
Follow‐up biopsy			
GGG 0	0 (0.0)	10 (17.5)	8 (12.1)
GGG 1	2 (2.7)	5 (8.7)	9 (13.6)
GGG 2	5 (6.8)	2 (3.5)	11 (16.7)
GGG 3–5	1 (1.4)	1 (1.8)	1 (1.5)

PI‐RADS = Prostate Imaging Reporting Data System v2.1; GGG = Gleason Grade Group.

Table 4 presents factors affecting the diagnosis of csPCa during follow‐up. A multivariate regression odds ratio (95% CI) was used. Baseline PI‐RADS finding was the only significant factor which associated with csPCa found during follow‐up.

**TABLE 4:** Factors Associated With the Diagnoses of Clinically Significant Prostate Cancer During the Follow‐Up

Characteristic	No csPCa in Follow‐Up‐Biopsies	csPCa in Follow‐Up‐Biopsies	Univariate Regression			Multivariate Regression		
			Odds Ratio	95% Cl	*P*	Odds Ratio	95% Cl	*P*
Baseline PI‐RADS score								
PI‐RADS 4–5 (Ref)	54 (31)	12 (57)	‐			‐	‐	‐
PI‐RADS 3	54 (31)	3 (14)	0.25	0.05–0.84	0.040	0.21	0.04–0.73	0.024
PI‐RADS 1–2	68 (39)	6 (29)	0.40	0.13–1.09	0.083	0.34	0.11–0.97	0.052
Age (years)	64 (60, 68)	63 (60, 66)	0.98	0.92–1.05	0.5	0.97	0.91–1.04	0.4
PSA density (ng/mL^2^)	0.15 (0.10, 0.22)	0.20 (0.13, 0.29)	0.86	0.21–5.82	0.8	0.69	0.16–4.43	0.6
5‐Alpha reductase therapy								
No (Ref)	147 (84)	16 (76)	‐			‐	‐	‐
Yes	29 (16)	5 (24)	1.58	0.49–4.42	0.4	2.26	0.66–6.94	0.2

Data are median (interquartile range) or number (percentage).

Cl = confidence interval; PSA = prostate specific antigen; PI‐RADS = Prostate Imaging Reporting Data System v2.1.

**FIGURE 2:**
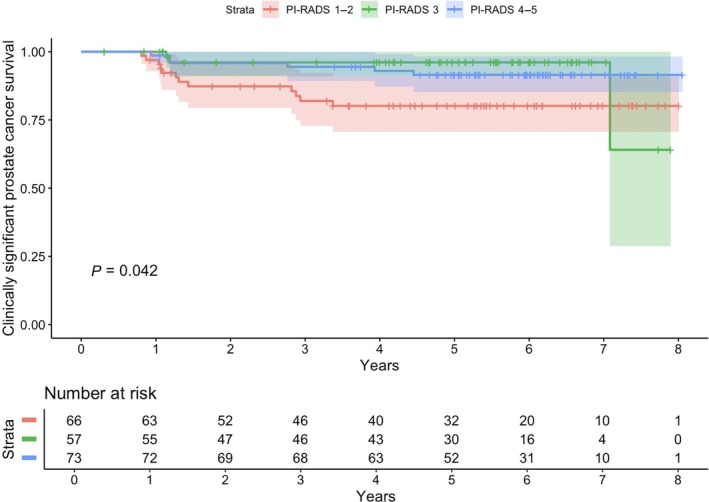
Clinically significant prostate cancer (csPCa) survival rate.

When baseline bpMRI had PI‐RADS scores of 1–2, the probability of csPCa was really low even with a follow‐up time of at least 6 years (Fig. 2). In this study, the probability of csPCa was equally low with baseline bpMRI PI‐RADS score of 3; the first csPCa case was detected in year 1, and the last two were identified in year 7. When baseline bpMRI had PI‐RADS scores of 4–5, the probability of csPCa found during follow‐up was relatively high, especially during the first 3 years of follow‐up.

## Discussion

Of the men with a baseline bpMRI PI‐RADS 1–3 finding and benign or GGG 1 PCa in the initial biopsies, only few men were diagnosed with GGG ≥2 PCa during the follow‐up. The number of csPCa being diagnosed in patients with PI‐RADS 1–2 in the baseline biopsies was a little higher than in men with PI‐RADS 3, but this is mostly due to a coincidence and relatively small sub‐cohorts. The follow‐up biopsies were taken by any urologists of the study center whereas the initial biopsies were taken only by a few specific urologists involved in the study. In addition, within men with bpMRI PI‐RADS 3 finding in initial bpMRI, csPCa was more likely already diagnosed in the baseline biopsies, since more biopsies were taken from PI‐RADS 3 lesions: both targeted biopsies and systematic biopsies. Moreover, of men with PI‐RADS 4–5 findings and GGG ≤1 PCa in the initial biopsies, significantly more men were diagnosed with GGG ≥2 PCa during the follow‐up. Finally, under 2% of the patients with baseline bpMRI PI‐RADS 1–3 were diagnosed with PCa of GGG >2.

When looking at the time to diagnosis of csPCa, a special exception is seen at the seventh year of follow‐up of bpMRI PI‐RADS 3 population, where a disproportionate number of csPCas were found. However, this is explained by very few patients at that point and therefore, any relevant conclusions cannot be drawn from that exception in line with such a small sample and long, over 6 years of follow‐up time. When baseline bpMRI had PI‐RADS scores of 4–5, the probability of csPCa found during follow‐up was relatively high, especially during the first 3 years of follow‐up.

There was no specific predesigned follow‐up plan for these patients, but it is very unlikely that any csPCa would have been missed due to the nature of the Finnish public healthcare system since all prostate cancer is managed at the same center as the biopsies are taken.

Only a few follow‐up studies concerning men with PI‐RADS 3 findings at the initial prebiopsy mpMRI have been published, with multiple variations in the study design.^15^, ^16^, ^17^, ^18^ It is therefore of clinical interest to see long‐term follow‐up results for men who underwent bpMRI with initial negative prostate biopsy. In the 60 months follow‐up study by Venderink et al (*N* = 4259) the detection rate for csPCa (GGG ≥ 2) after negative biopsies (GGG ≤ 1) and PI‐RADS 3 mpMRI findings (*N* = 110) was 6.4%.^15^ The long‐term follow‐up results of the retrospective study by Önder et al (*N* = 1359) shows that csPCa (GGG ≥ 2) rate in men with a PI‐RADS 3 finding in mpMRI and negative biopsies was 5.4% in a 5‐year follow‐up period.^16^ These are in line with the results in this study where the corresponding rate with bpMRI is 5.3%; hence, the risk of being diagnosed with csPCa is almost as low as with a prebiopsy bpMRI PI‐RADS score of 1–2. In addition to these studies at least two studies have taken into account the follow‐up of the mpMRI PI‐RADS 3 findings but with a relatively short follow‐up time and with quite different study settings; the rates of GGG ≥2 ranged from 4% to 4.5% in these studies.^17^, ^18^ Comparing bpMRI and mpMRI, some of the PI‐RADS 3 lesions were upgraded to PI‐RADS 4 due to contrast enhancement, thus these lesions were interpreted as PI‐RADS 3 lesions with bpMRI. Despite this, as seen in this current analysis, the detection rate for csPCa in the follow‐up with bpMRI does not seem to be higher compared to mpMRI.

The current EAU guidelines recommend sharing decision‐making with patients when omitting a biopsy in a patient with a negative MRI (i.e., PI‐RADS ≤ 2).^1^, ^24^ This policy is further supported by the data of this study and previous studies which demonstrate that a negative bpMRI (PI‐RADS score 1–2) has a high negative predictive value for the detection of aggressive csPCa in a long‐term follow‐up.^13^, ^14^, ^15^, ^16^ In a retrospective follow‐up analysis of the Biparametric MRI for Detection of Prostate Cancer (BIDOC) trial, only 1.7% (5/301) of the subjects with a bpMRI PI‐RADS score of 1–2 presented with csPCa (GGG ≥ 3 or a maximum cancer core length greater than 50% of GGG 2) during the 5 years of follow‐up.^13^ In addition, in a follow‐up analysis of the FUTURE trial, the risk of being diagnosed with csPCa (GGG ≥ 2) after negative (PI‐RADS 1–2) mpMRI and negative systematic biopsies was 3% in the median follow‐up time of 44 months.^14^ These studies corroborate the results of this study where under 2% of patients with PI‐RADS 1–3 were diagnosed with GGG ≥3. Furthermore, in two additional studies with varied study settings, the detection rate of csPCa within 5–6 years after negative PI‐RADS 1–2 mpMRI ranged from 5.9% to 6.2%, which is in line with the results of this current study.^15^, ^16^

The scenario is very different as regards the baseline MRI PI‐RADS 4–5 findings. According to the study by Schoots et al the prevalence of GGG ≥2 PCa is more than 50% in these cases, and EAU guidelines state that prostate biopsies should always be taken.^1^, ^25^ However, even with MRI PI‐RADS 4–5 findings sometimes biopsies give negative results. Targeting errors do occur, and therefore, early repeated mpMRI and repeated prostate biopsies are recommended among men with PI‐RADS 4 or 5 findings without csPCa on the initial biopsy but persistent PI‐RADS >2 scores in MRIs, as recommended by the prospective study of Becher et al.^26^ In addition, Hauth et al demonstrated that in men with mpMRI PI‐RADS 4 finding and with negative initial biopsies, the rate of csPCa was as high as 69%.^22^ In this retrospective study, men with bpMRI PI‐RADS scores 4–5 but benign or GGG 1 in the initial biopsies, had 18% risk of being diagnosed with GGG ≥2 PCa within the follow‐up time, which supports more rigorous follow‐ups for these men.

The rate of incident of GGG ≥2 PCa during a follow‐up has been described in four studies, three utilizing mpMRI and one bpMRI.^13^, ^14^, ^15^, ^16^ When these rates are compared between different studies, it should be acknowledged that the baseline PI‐RADS distribution heavily affects the results. The distribution is of course affected by the incidence of PCa in the study population but also the experience of the radiologist. Although the PI‐RADS guidelines have set standards for prostate MRI reporting, it is well known that the interpretation is reader‐dependent. In the previous studies, the rates of baseline PI‐RADS 1–2 findings ranged from 28% to 65%, PI‐RADS 3 findings ranged from 10% to 35%, and PI‐RADS 4–5 findings ranged from 26% to 57%.^6^, ^15^, ^16^, ^27^ The rates of baseline PI‐RADS 3 findings in most of the reported prior studies roughly correspond to the rates in this current study (15%), whereas in the current study significantly more PI‐RADS 4–5 findings (66%) were observed at the baseline biopsies.

### Limitations

This is a retrospective, single center analysis of prospectively collected data without a prespecified follow‐up protocol. Due to the nature of the Finnish healthcare system, where all PCa are managed at the same center where biopsies are performed, csPCa is unlikely missing in the cohort. Men with PI‐RADS 1–3 are not followed up as rigorously as men with PI‐RADS 4–5 findings, but all men with GGG 1 in the initial biopsies stay under surveillance in the study center. Men with benign biopsy results, which are not surveyed in the study center, are referred back in case of a remaining suspicion of PCa (i.e., rising PSA value or abnormal finding in digital rectal examination). The study cohort, for example, men with baseline bpMRI PI‐RADS score of 3, is relatively small (*N* = 57), which is responsible for the abrupt drop in Fig. 2. In addition, we did not evaluate inter‐reader variability since all MRI reading was reviewed by a central radiologist who made the final decision in case of disagreement. Therefore, the results may not be generalized to other centers. Finally, the study population in Finland differs from other Western countries because of the low rate of opportunistic PSA screening, which most probably increases the rate of baseline PI‐RADS 4–5 findings as seen in this retrospective study.

## Conclusions

The study showed that in men with bpMRI‐based PI‐RADS score of 1–3 and the initial negative prostate biopsy for csPCa, the risk of being diagnosed with csPCa during follow‐up is very low, thus supporting more conservative follow‐up for these men. However, for men with PI‐RADS 4–5 findings a more rigorous follow‐up should be initiated.

## Supporting information


**Table S1.** Characteristics of follow‐up procedures in subjects separated into two subgroups; men with no cancer and men with GGG 1 cancer at baseline biopsies.
