# Supplementary material for: Long‐Term Risk of Clinically Significant Prostate Cancer in Biopsy‐Negative Patients With Baseline Biparametric Prostate MRI
Source: J Magn Reson Imaging. 2024 Nov 27;61(6):2425–32. doi: 10.1002/jmri.29668 (PMC12063762; doi:10.1002/jmri.29668)
Supplement: Supplementary file 1 — Table S1. Characteristics of follow‐up procedures in subjects separated into two subgroups; men with no cancer and men with GGG 1 cancer at baseline biopsies. [file JMRI-61-2425-s001.docx]

Supplementary Table 1

Supplementary Table 1. Characteristics of follow-up procedures in subjects separated into two subgroups; men with no cancer and men with GGG1 cancer at baseline biopsies

| **Baseline MRI** | **PI-RADS 1-2 n=74** | | **PI-RADS 3 n=57** | | **PI-RADS 4-5 n=66** | |
| --- | --- | --- | --- | --- | --- | --- |
| **Baseline biopsies**  **GGG 0, GGG 1** | **GGG 0 n= 62** | **GGG 1 n=12** | **GGG 0 n=42** | **GGG 1 n=15** | **GGG 0 n=33** | **GGG 1 n=33** |
| **FU time** | 34 (14-53) | 77 (62-81) | 32 (20-62) | 71 (62-80) | 53 (19-76) | 63 (38-84) |
| **FU PSA*** | 52 (84.0) | 12 (100.0) | 40 (95.0) | 15 (100.0) | 30 (91.0) | 33 (100.0) |
| **FU PSA**** | 2 (1-4) | 14 (13-17) | 4 (3-7) | 15 (9-17) | 4 (2-8) | 13 (9-16) |
| **FU MRI*** | 8 (13.0) | 7 (58.0) | 12 (29.0) | 6 (40.0) | 13 (39.0) | 16 (49.0) |
| **FU Bx*** | 1 (2.0) | 7 (58.0) | 9 (21.0) | 9 (60.0) | 8 (24.0) | 21 (64.0) |
| * Number and proportion of subjects with follow-up PSA, MRI and prostate biopsies | | | | | | |
| ** Median number of follow-up PSA measurements in individual subjects | | | | | | |
| Data are median (interquartile range) or number (percentage).  FU, follow-up; m, month; PSA, prostate specific antigen, MRI, magnetic resonance imaging; Bx, prostate biopsy; PI-RADS, Prostate Imaging Reporting Data System v2.1; GGG, Gleason Grade Group | | | | | | |
|  | | | | | | |
|  | | | | | | |
